# NeuroPredict: study of the predictive value of ABCB1 genetic polymorphisms and associated clinical factors in chronic chemotherapy-induced peripheral neuropathy (CIPN)

**DOI:** 10.3389/fphar.2024.1352939

**Published:** 2024-02-26

**Authors:** Alicia Vargas-Aliaga, María De la Haba, María José Contreras, Cristina Morales Estevez, Ignacio Porras, María Teresa Cano, Gema Pulido, María Auxiliadora Gómez, Pablo Flores-Paco, De La Haba-Rodriguez Juan, Enrique Aranda

**Affiliations:** ^1^ Hospital Universitario Reina Sofía Córdoba, Córdoba, Spain; ^2^ Instituto Maimónides de Investigación Biomédica de Córdoba (IMIBIC), Córdoba, Spain; ^3^ Universidad de Córdoba, Córdoba, Spain

**Keywords:** chronic neuropathy, chemotherapy, polymorphism, ABCB1, risk factors

## Abstract

**Background:** Chemotherapy-induced peripheral neuropathy (CIPN) is a common entity (30%–40%) and can significantly limit the quality of life of patients, especially those that persist for more than 6 months after treatment (chronic neuropathy). Studies have shown a possible association between the presence of genetic polymorphisms in ABCB1 and the development of acute CIPN, although this relationship with chronic CIPN remains unexplored. This is an analytical observational case-control study defined by the presence (cases) or absence (controls) of CIPN at 6 months after the end of the neurotoxic drug. Our aim is to demonstrate whether these ABCB1 polymorphisms also influence the chronification of this toxicity, as well as the clinical factors that can help us to predict it.

**Methods:** The study included 152 patients treated with tri-weekly oxaliplatin (O) or weekly paclitaxel (P); 86 cases and 66 controls. Clinical and analytical parameters were analysed including the study of ABCB1 genetic polymorphisms in a blood sample.

**Results:** ABCB1 genetic polymorphisms C1236T (rs1128503) and C3435T (rs1045642) are associated with the development of chronic CIPN in patients treated with P. No differences were found in patients treated with O. Other predictive factors to be considered in the development of this toxicity are age >60 years, BMI ≥30, toxic habits and cardiovascular risk factors.

**Conclusion:** CIPN is a common and understudied toxicity, despite being a limiting factor in the quality of life of many patients. As described in acute CIPN, our study demonstrates the relationship between chronic neuropathy and being a carrier of specific polymorphisms (C1236T and C3435T) of the ABCB1 gene in patients treated with P. In addition, there are modifiable factors (obesity, smoking, or alcohol) that may influence its development. Further prospective studies are needed to investigate genetic and clinical modifiable factors predisposing to CIPPN to develop prevention and treatment strategies.

## Introduction

Chemotherapy is one of the most widely used therapeutic modalities in the treatment of cancer. The function of this group of drugs is to stop cell growth and division, by acting at multiple control points in the cell cycle, to control the disease (DeVita et al., 2001).

However, most chemotherapy drugs have a major limitation: low specificity and a narrow therapeutic margin. Their mechanism of action is to cause cellular alterations, which, although varying according to the type and dose at which they are administered, also affect other normal cells and tissues in the body, especially those undergoing constant active division. For this reason, toxicity becomes one of the limiting factors in the use of this antineoplastic therapy (Rosell et al., 2002).

Chemotherapy-induced peripheral neuropathy (CIPN) is the most frequent neurological complication of cancer treatment and it is probably the most common toxic neuropathy in our environment (Rosell et al., 2002) (1) 30%–40% of all patients treated with chemotherapeutic agents develop peripheral neurotoxicity (Hu et al., 2018), and up to 60% with paclitaxel or oxaliplatin (Seretny et al., 2014). It is a progressive, sometimes chronic, and irreversible side effect produced by some antineoplastic drugs (Hu et al., 2018). It has been estimated that over half the patients treated with paclitaxel experience symptoms. Approximately 50% of patients who developed CIPN recover within 4–6 months, although severe neuropathy may persist for years (Tanabe et al., 2017). On the other side, almost 90% of patients who received treatment with oxaliplatin develop the acute neuropathy and it has been estimated that approximately 70% will suffer chronic CIPN (Grothey, 2005).

With more and more patients surviving cancer, CIPN is becoming an issue of public health concern, as its appearance leads to delays in the administration of cycles, dose reductions or even suspension of treatment, thus affecting the curative potential of treatment and the patient’s prognosis (Velasco and Bruna, 2010).

Paclitaxel-induced peripheral neuropathy predominantly presents with sensory symptoms without motor symptoms (Kus et al., 2016). On the other hand, acute oxaliplatin-induced neuropathy is characterized by dysesthesia and paresthesia of the hands and feet, and motor symptoms (like tetanic spasms or fasciculations) can also be present. These symptoms are often exacerbated by cold. Clinically, the symptoms of chronic oxaliplatin-induced neuropathy closely resemble those of the acute form but additionally, changed proprioception negatively affecting normal daily activities that require fine motor coordination might also occur (Grothey, 2005). In comparison to other peripheral neuropathies, for example, painful diabetic polyneuropathy, patients with CIPN may present more fulminant symptoms, affecting at the same time the feet and hands, with predominant pain, and symptoms have a faster progression as well. According to findings coming from electrodiagnostic studies, CIPN may be characterized as an axonal sensorimotor neuropathy, while painful diabetic neuropathy may be classified as a mixed neuropathy (Azhary et al., 2010).

Although the exact molecular mechanism that produces this peripheral neuropathy remains poorly understood, it is thought that it may be caused by direct damage to the peripheral nervous system (PNS), leading to these sensory symptoms.

In recent years, clinical and experimental research has increased to understand the pathophysiology, prevention, and treatment of this pathology, although nowadays early recognition and initial management are the best and only techniques available to avoid progression to severe and disabling neuropathy (Tang et al., 2019). Once established, CIPN has no effective treatment. Depending on the dose and the agent used, symptoms can only sometimes resolve completely (acute neuropathy). However, neurotoxicity is often only partially reversible, and in some cases, irreversible and very limiting (chronic neuropathy) (Azhary et al., 2010). As reviewed in the literature, the prevalence of CIPN after the first month of starting chemotherapy was 68.1%, 60% at 3 months and 30% at 6 months or more (Seretny et al., 2014). It is the latter clinical group that is of most interest and on which this study is focused.

Many factors influence the variability of CIPN: the type of cytostatic agent and treatment schedule administered (total dose, number of cycles, infusion time), combinations of different cytostatic, the type of tumour, concomitant use of other neurotoxic drugs, and the criteria or technique of diagnosis and assessment of CIPN (Wen, 2003). The association of clinical risk factors that may predict the development of acute neuropathy has also been described, such as diabetes, high body mass index, consumption of toxic substances (tobacco or alcohol), in older patients or those with renal failure, and also those patients with a history of previous liver disease, a history of previous neuropathy or even low hemoglobin levels in the blood (Seretny et al., 2014). Recently, possible biomarkers predictive of this pathology have been studied.

Paclitaxel is a key chemotherapeutic agent in the treatment of early-stage and metastatic breast cancer. Paclitaxel is transported into hepatocytes by solute carrier organic anion transporter family member 1B3 (SLCO1B3) and is metabolized by cytochrome P450 (CYP450) in the liver (Guijosa et al., 2022). Some of the genes which are involved in transport through hepatobiliary and intestinal secretion of the drug are the adenosine thriphosphate-binding cassette (ABC) genes, ABCB1 ABCC1 and ABCC2 isoforms. Elimination pathway of taxanes is mediated by the membrane-localized, energy-dependent drug efflux ABC transporter, P-glycoprotein (P-gp), which has been described in several polymorphisms. Treatment. It was reported that there is a possible relation-ship between single nucleotide polymorphisms (SNPs) of the ABCB1 gene, which affect the activity of P-gp, and the clinical outcome and toxicity of patients who were treated with taxane (Kus et al., 2016; de Castro et al., 2019).

In terms of oxaliplatin-induced peripheral neuropathy, the severe chronic entity has been reported in up to 18% of oxaliplatin-treated CRC patients included in phase III clinical studies and its incidence is dose-cumulative, due to accumulation of platinum adducts inside dorsal root ganglia (DRG) and peripheral neurons (Grothey, 2005). Some studies have determined that the pathophysiology of the acute entity sensory disturbances produced by this drug are the result of a transient alteration of ion channels and nerve hyperexcitability due to sodium channel activation (Gamelin et al., 2004). On the other hand, to date, several studies have investigated single nucleotide polymorphisms (SNPs) involved in oxaliplatin DNA transport, detoxification and repair (Seretny et al., 2014). Furthermore, the regulation of oxaliplatin detoxification and transport is polyallelic. Indeed, several SNPs may be associated with altered expression or function of gene products involved in both extracellular and intracellular transport, leading to increased oxaliplatin concentrations in the DRG (Stage et al., 2021).

Other studies describe associations with genetic variants of these outward transporters with functional modifications that are associated with an increased risk of peripheral neuropathy. This may be due to the accumulation of drugs in the PNS due to a reduction in the neuronal capacity to expel them.

There are numerous genetic factors that could influence the variability of neurotoxicity, but we focus on ABCB1 polymorphisms, specifically three of them because they are the most important in bioavailability and limiting the toxicity in the cell of a wide range of drugs and xenobiotics (Chen et al., 2021; Yan et al., 2022).

This line of research is therefore aimed at clarifying whether there is a relationship between the development of chronic CIPN and ABCB1 polymorphisms, thus contributing to establishing a strategy for prevention and counselling of patients predisposed to this limiting toxicity.

## Methods

We performed a single center, analytical observational case-control study in patients diagnosed with breast and colorectal cancer for whom treatment with paclitaxel (80mg/m2/weekly) or oxaliplatin (130 mg/m2/every 3 weeks), respectively, was indicated. All included patients have received treatment with the neurotoxicant as adjuvant treatment and for 18 weeks for oxaliplatin and 8–12 weeks for paclitaxel.

**FIGURE 1:**
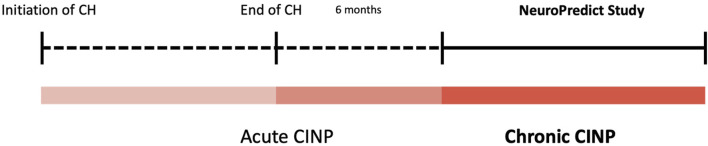
Location in time of NeuroPredict study.

Both following groups were defined: Cases/Group A: Presence of Neuropathy 6 months after the end of the neurotoxic drug (Chronic Neuropathy). Controls/Group B: No neuropathy 6 months after the end of the neurotoxic drug (Figure 1).

The study was performed between September 2022 and May 2023 at the Medical Oncology Service at the University Hospital Reina Sofia in Córdoba, Spain. The Medical Ethics Committee and the board of directors of the Hospital approved the study protocol.

The study was performed in accordance with the International Conference on Harmonization Good Clinical Practice guidelines, the Declaration of Helsinki, and all applicable regulations. All patients provided written informed consent before any study related procedure was pursued.

### Patients

Patients, 18 years or older, were eligible if they were diagnosed with an early-stage breast or colorectal cancer. They were treated with different adjuvant chemotherapy with paclitaxel (P) (80mg/m2/weekly) in monotherapy or oxaliplatin (O) (130 mg/m2/every 3 weeks) with capecitabine (1000mg/m2/12 h 14 days; XELOX scheme), respectively, and they were required to be disease-free at the time of the study. Patients had to have an Eastern Cooperative Oncology Group (ECOG) performance status of 0 or 1. Patients had to have the presence of chronic neuropathy at least grade 1, for group A (cases) and absence of chronic neuropathy for group B (controls).

### Study design

The hypothesis of our study was: “Polymorphisms in the ABCB1 gene predict the development of chronic CIPN secondary to treatment with paclitaxel and oxaliplatin.” The main objective of the study was to determine whether there is an association between the development of chronic CIPN after oncological treatment with oxaliplatin or paclitaxel and polymorphisms in the ABCB1 gene. Secondary objectives focused on study the association between clinical and analytical factors and the development of chronic CIPN; and on develop a predictive model of chronic CIPN risk considering clinical factors.

All data were collected from two sources: electronic medical records registry (^®^Diraya), in which clinical data and background information of interest were recorded; and ^®^OncoFarm software from which data were extracted regarding the drug, dosage and possible reductions or suspensions in treatment.

All included patients were seen at the outpatient clinic, where they signed the corresponding informed consent form. Those who wished to participate in the study had a blood sample taken at the Clinical Research Unit of Medical Oncology.

### Genetic polymorphisms of ABCB1

To study the genetic polymorphisms of ABCB1 as possible predictors of chronic CIPN, three types of analysis were carried out. Firstly, an individualised analysis was carried out to see which of these polymorphisms had the greatest influence on our main variable. Secondly, a grouped analysis was performed according to the polymorphism with the greatest weight. For the remaining polymorphisms, two groups were formed. Finally, we distinguished the polymorphisms according to the chemotherapy drug the patients had received, to assess whether there was a difference between them.

### Genotyping analysis

DNA was extracted from nucleated cells present in 5 mL peripheral blood samples in EDTA tubes using an automated DNA extractor (MagNA Pure^®^ System, Roche Applied Science, Indianapolis, Indiana) and quantified by spectrophotometry on a NanoDrop^®^ ND-1000 spectrophotometer (Wilmington, USA). Genotyping and allelic discrimination of ABCB1 was performed with a QuantStudio 12k Flex PCR-RT system (Thermo Fisher Scientific, Massachusetts, USA) and Sanger sequencing. The following Single Nucleotide Polymorphisms (SNPs) rs1128503, rs2032582 and rs1045642, located on chromosome 7, were analysed.

### Statistical analysis

#### Sample size calculation

**FIGURE 2:**
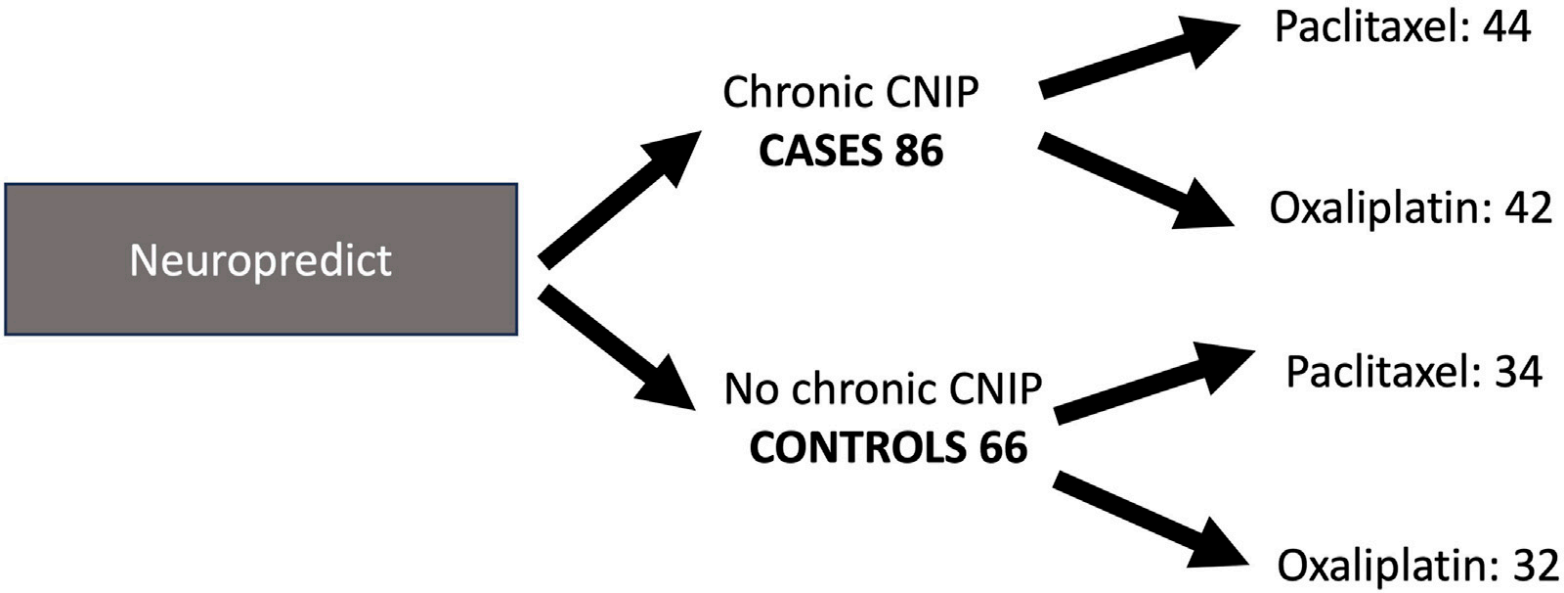
Sample distribution. Neuropredict study.

Sample size calculation was performed with the GRANMO software (v7.12 April 2012). We calculate the sample size with the proposed established assumptions: Accepting an alpha risk of 0.05 and a beta risk of 0.2 in a bilateral contrast, 64 subjects in the first group and 32 in the second are needed to detect a difference equal to or greater than 0.3 between the two. A ratio of 0.3 is assumed in one of the groups. A loss-to-follow-up rate of 0.01 was estimated. The ARCOSENO approximation was used. In this study, a total of 152 patients have been enrolled: 86 cases (44 treated with paclitaxel and 42 treated with oxaliplatin) and 66 controls (Figure 2).

i. Variables

Chronic neuropathy was used as the dependent variable. This variable was considered dichotomously (presence/absence). As independent variables, possible clinical factors associated with neuropathy were included in the analysis (haemoglobin (Hb) and creatinine clearance (ClCr) at the start of treatment, cardiovascular risk factors (high blood pressure (HBP), diabetes mellitus (DM) or high body mass index (BMI)), toxic habits (smoking or drinking), sex, age, drug used, treatment schedule and dose, as well as the analysis of the three ABCB1 polymorphisms. For the descriptive analysis of the clinicopathological characteristics of the patients, we used quantitative and qualitative variables, described in Table 1.

**TABLE 1:** Descriptive analysis of the data. Qualitative and quantitative variables.

Qualitative variables	Category	Absolute frequency (%)			
		Paclitaxel cases (n = 44)	OXALIPLATIN cases (n = 42)	Paclitaxel controls (n = 34)	OXALIPLATIN controls (n = 32)
SEX	Male	1 (2,3)	26 (61,9)	1 (2,9)	19 (59,4)
	Female	43 (97,7)	16 (38,1)	33 (97,1)	13 (40,6)
AGE	<60 years old	24 (54,5)	8 (19)	26 (76,5)	13 (40,6)
	≥60 years old	20 (45,5)	34 (81)	8 (23,5)	19 (59,4)
BMI	<30	28 (63,6)	28 (66,7)	30 (88,2)	26 (81,3)
	≥30	16 (34,6)	14 (33,3)	4 (11,8)	6 (18,8)
PREVIOUS ACUTE NEUROPATHY	Yes	44 (100)	42 (100)	11 (32,4)	19 (59,4)
	No	0 (0)	0 (0)	23 (67,6)	13 (40,6)
TOXIC HABITS	Smokes	12 (27,3)	17 (40,5)	6 (17,6)	5 (15,6)
	Drinks alcohol	1 (2,3)	6 (14,3)	1 (2,9)	3 (9,4)
	Smokes and drinks	3 (6,8)	6 (14,3)	0 (0)	2 (6,3)
	Neither smokes nor drinks	28 (63,6)	13 (31)	27 (79,4)	22 (68,8)
CARDIOVASCULAR RISK FACTORS (HTA/DM/dyslipidemia)	Yes	11 (25)	24 (57,1)	2 (5,9)	13 (40,6)
	No	33 (75)	18 (42,9)	32 (94,1)	19 (59,4)
CLCR FIGURES	Normal (>60 mL/min)	43 (97,7)	34 (81)	34 (100)	30 (93,8)
	Anormal (<60 mL/min)	1 (2,3)	8 (19)	0 (0)	2 (6,3)
HAEMOGLOBIN FIGURES	Normal (Male >14 g/dL; female >12 g/dL)	37 (84,1)	26 (61,9)	26 (76,5)	25 (78,1)
	Anormal (Male <14 g/dL; female <12 g/dL)	7 (15,9)	16 (38,1)	8 (23,5)	7 (21,9)
CHRONIC LIVER DISEASE	Yes	1 (2,3)	3 (7,1)	0 (0)	1 (3,1)
	No	43 (97,7)	39 (92,9)	34 (100)	31 (96,9)
QUANTITATIVE VARIABLES		Mean (SD)			
		**PACLITAXEL CASES (n = 44)**	**OXALIPLATIN CASES (n = 42)**	**PACLITAXEL CONTROLS (n = 34)**	**OXALIPLATIN CONTROLS (n = 32)**
	Age	59,04 (9,70)	64,51 (7,12)	56,74 (7,29)	62,14 (9,92)
	BMI	27,87 (5,54)	28,16 (4,87)	25,79 (4,47)	27,31 (3,98)

SD: standard deviation.

#### Procedure

For the statistical analysis, a descriptive study was first performed by calculating absolute frequencies for qualitative variables, and arithmetic mean, median, standard deviation and variance for quantitative variables. A 95% confidence interval was estimated.

For the bivariate analysis, the Chi-square statistic was used to compare qualitative variables.

For the development of the score, a multivariate analysis was performed by selecting those variables of described clinical importance and those that reached a statistical significance of less than 0.2. As these were nominal quantitative variables, multiple logistic regression was used. For multiple comparisons, the Bonferroni test was used to correct the *p*-value.

All contrasts were bilateral and those where the *p*-value was less than 0.05 were considered significant.

Data were collected, processed, and analysed with the SPSS v.23 statistical software.

#### Ethical considerations

This project is subject to the standards of good clinical practice and was conducted in accordance with the principles set out in the Declaration of Helsinki. It was approved by the provincial research ethics committee of the Hospital Universitario Reina Sofía de Córdoba. The data were anonymized, respecting the confidentiality of the data, in accordance with Regulation (EU) 2016/679 of the European Parliament and Organic Law 3/2018, of 5 December, on Personal Data Protection and guarantee of digital rights.

## Results

### Genetic polymorphisms of ABCB1

1. Polymorphism C1236T (*rs1128503*)

a. Individualised analysis

For the individualised analysis, a 2xK cross-tabulation was performed. No statistically significant differences were observed between the three genotypes and the development of chronic CIPN. Among all patients who developed CIPN, 18.6% of patients had the T/T. However, of those who do not develop CIPN, 19.7% carry the T/T genotype. 41.9% of CIPN patients have T/C, compared to 53% of the control group. Of those with C/C genotype, 39.5% of patients had chronic CIPN compared to 27.3% who did not (X2 = 2.662, *p* = 0.264).

b. Group analysis

Despite not finding statistically significant differences in the individualized analysis of genotypes, we can intuit that the C/C variant is the one that produces the greatest difference in the incidence of chronic CIPN. We therefore grouped according to this variant *versus* the rest. No statistically significant conclusions were drawn from this analysis either (X2 = 2.495, **p = 0.114).**

c. Drug-specific analysis

When stratified according to the neurotoxic drug used, statistically significant differences were observed for patients treated with paclitaxel (X2 = 4.434, **p = 0.035)** (Table 2).

**TABLE 2:** Triple polymorphism C1236T (E12) analysis (OXL: oxaliplatin; PAC: Paclitaxel).

C1236T analysis		Genotype	CIPN chronic +	CIPN chronic - (%)	P
**Individual**		**T/T**	18.60%	19.70	0.264
		**T/C**	41.90%	53	
		**C/C**	39.50%	27.30	
**Grouped**		**C/C**	39.50%	27.30	0.114
		**T/T o T/C**	60.50%	72.70	
**According to drug**	**OXL**	**C/C**	28.60%	28.10	0.966
		**T/T o T/C**	71.40%	71.90	
	**PAC**	**C/C**	50%	26.50	**0.035**
		**T/T o T/C**	50%	73.50	

OXL, oxaliplatin; PAC, paclitaxel.

**FIGURE 3:**
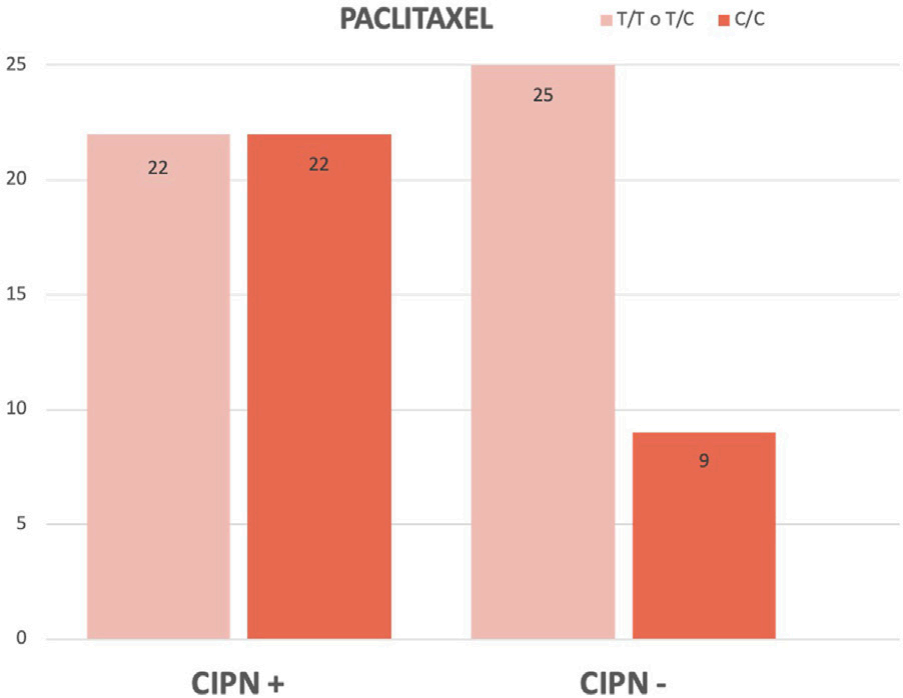
Comparison (in absolute frequency) between C1236T (E12) genotypes in case and control groups of paclitaxel-treated patients.

It could be argued that patients with T/T or T/C in the *G2677 T/A* polymorphism have less chronic CIPN. In other words, not being a carrier of the C/C variant protects against the development of this toxicity (Figure 3).

No statistically significant differences were established in those patients treated with oxaliplatin (X2 = 0.002, *p* = 0.996).

2. Polymorphism G2677 T/A (rs2032582)

a. Individualised analysis

No statistically significant differences were observed between the four genotypes with respect to the development of chronic CIPN (X2 = 2.623, *p* = 0.454).

b. Grouped analysis

In the individual analysis, we observed that the G/G variant produced the greatest difference in the incidence of chronic CIPN. We therefore grouped according to this variant *versus* the rest, although we also did not obtain statistically significant conclusions (X2 = 1.122, *p* = 0.289).

c. Drug-specific analysis

We stratified according to the drug used, and no statistically significant differences were observed for either oxaliplatin (X2 = 0.317, *p* = 0.574) or paclitaxel (X2 = 0.862, *p* = 0.353) (Table 3).

**TABLE 3:** Triple polymorphism G2677 T/A analysis.

G2677 T/A analysis		Genotype	CIPN chronic +	CIPN chronic -	P
Individual		T/T	16.30%	13.60%	0.454
		T/G	47.70%	54.50%	
		G/A	2.30%	6.1	
		G/G	33.70%	25.80%	
Grouped		G/G	33.70%	25,8%%	0.289
		Others	66.30%	74.20%	
According to drug	OXL	G/G	31%	25%	0.574
		Others	69%	75%	
	PAC	G/G	36.40%	26.50%	0.353
		Others	63.60%	73.50%	

3. Polymorphism C3435T (rs1045642)

a. Individualised analysis

We did a 2xK cross-tabulation with more than two groups and obtained a statistically significant result (X2 = 8.179, *p* = 0.017). However, when doing the table with more than two groups, we must compare two by two using a 2 × 2 cross-tabulation in order to draw conclusions. To do so, we did the following analysis.

b. Group analysis

In the analysis, the genotype with the most differences is the C/C genotype. Therefore, we separated into two groups: C/C compared to the rest of the genotypes. Statistically significant differences are observed (X2 = 6.992, *p* = 0.008) (Table 4).

**TABLE 4:** Triple polymorphism C3435T analysis.

C3435T analysis		Genotype	CIPN chronic +	CIPN chronic - (%)	P
Individual		T/T	36%	21.20	0.017
		T/C	47.70%	43.90	
		C/C	16.30%	34.80	
Grouped		C/C	16.30%	34.80	0.008
		T/T o T/C	83.70%	65.20	
According to drug	OXL	C/C	19%	34.40	0.135
		T/T o T/C	81%	65.60	
	PAC	C/C	13.60%	35.30	0.024
		T/T o T/C	86.40%	64.70	

**FIGURE 4:**
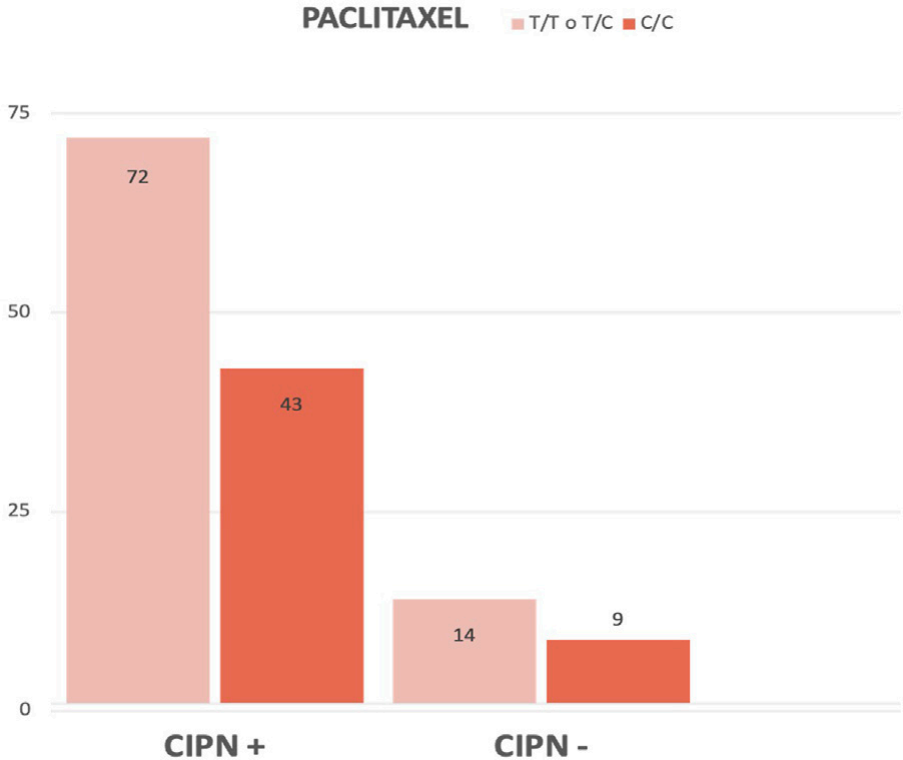
Comparison (in absolute frequency) between the pooled C3435T genotypes in the case and control groups.

As shown in Figure 4, we can conclude that the proportion of chronic CIPN is lower in patients with the C/C genotype of the *C3435T* polymorphism than in patients with the other variants.

c. Drug-specific analysis

**FIGURE 5:**
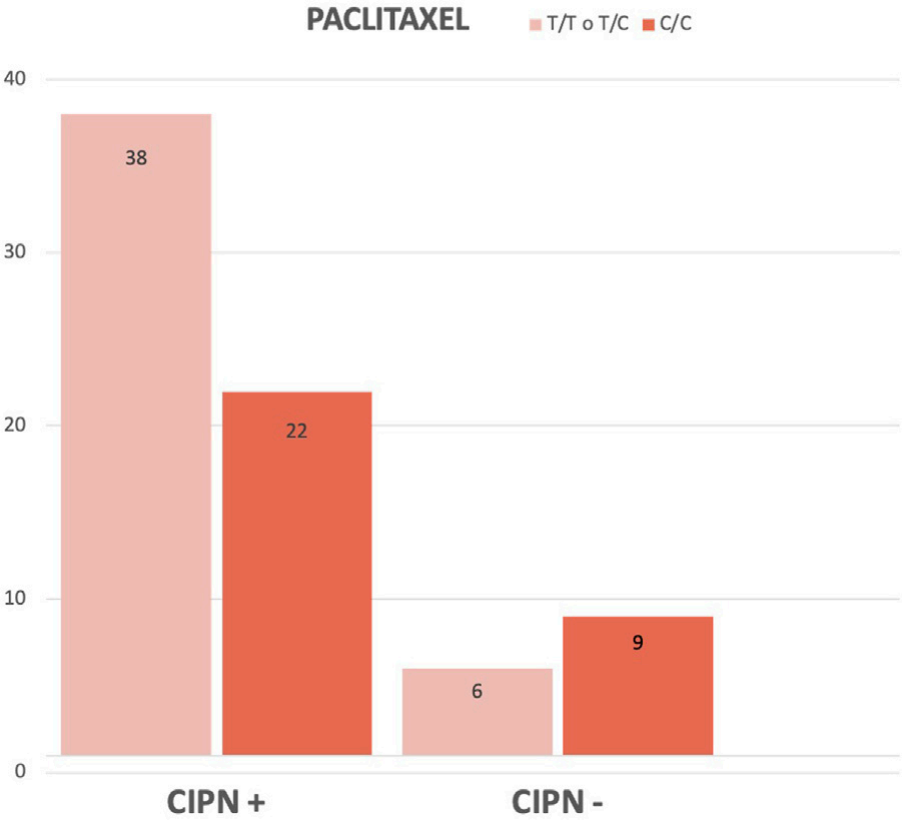
Comparison (in absolute frequency) between C3435T genotypes in the case and control groups of Paclitaxel-treated patients.

When divided according to neurotoxicant received, we found no statistically significant differences in patients treated with oxaliplatin (X2 = 2.236, *p* = 0.135), but did find statistically significant differences in patients treated with paclitaxel (X2 = 5.068, *p* = 0.024) (Figure 5).

We can state that the percentage of paclitaxel-treated patients who develop chronic CIPN is lower in the group of patients with the C/C genotype.

### Other related factors

In addition to analysing genetic factors, clinical and analytical factors were studied which, according to the existing literature, could be related to the development of chronic CIPN (Wen, 2003; Tang et al., 2019). The results are shown in Table 5. We can observe that those patients over 60 years of age, with a BMI greater than or equal to 30, who have a toxic habit (tobacco and/or alcohol) and who suffer from some CVRF such as hypertension, diabetes mellitus or hypertriglyceridemia (one or more) develop a higher proportion of chronic CIPN, with statistically significant results. In relation to previous chronic liver disease, Hb and CrCl at the start of treatment, no statistically significant results were obtained.

**TABLE 5:** Analysis of other factors associated to chronic CIPN.

Potential associated risk factors		Proportion		p
		Chronic CIPN +	Chronic CIPN - (%)	
AGE	<60 years old	45.10%	54.90	0.007
	>60 years old	66.70%	33.3	
BMI	<30	50%	50	0.006
	>30	75%	25	
TOXIC HABITS	Smoking and/or drinking	72.60%	27.40	<0.001
	No smoking or drinking	45.60%	54.40	
CARDIOVASCULAR RISK FACTORS	Yes	70%	30	0.019
	No	50%	50	
PREVIOUS CHRONIC LIVER DISEASE	Yes	80%	20	0.283
	No	55.80%	44.20	
HB FIGURES AT BASELINE	Normal	55.30%	44.70	0.571
	Anormal	60.50%	39.50	
ClCr FIGURES AT BASELINE	Normal	54.60%	45.40	0.08
	Anormal	81.80%	18.20	

### Risk score. multiple logistic regression

#### Whole sample (only clinical factors)

Considering chronic CIPN as the main variable, we constructed a multiple logistic regression model that can help us predict whether the patient will suffer from it or not, depending on the different covariates.

ABCB1 genetic polymorphisms were not included in the multivariate analysis of the whole sample as we only found statistically significant differences for the development of chronic CIPN with the paclitaxel-treated population for two of the polymorphisms. Therefore, we included only clinical factors in this first analysis.

First, univariate analyses were performed to show how each covariate is related to the main covariate in isolation, and those with *p* < 0.25 were selected.

- Age greater than or equal to 60: 2.44 times higher risk of chronic CIPN (p:0.008).
- BMI greater than or equal to 30: 3-fold increased risk of chronic CIPN (p: 0.008).
- Consumption of at least one intoxicant habit (tobacco and/or alcohol): 3.16 times higher risk of chronic CIPN (p:0.001)
- Presence of at least one cardiovascular risk factor (diabetes mellitus, hypertension and/or dyslipidaemia): 2.33 times higher risk of chronic CIPN (p:0.021).

A multiple logistic regression analysis was then performed. We first obtained a larger model with all the variables, from which those with *p* > 0.15 were extracted from the largest to the smallest. A smaller model was then obtained, with the following variables: age over 60 years, BMI over 30 and presence of at least one toxic habit. In this model it was found.

- Interaction between age over 60 and BMI over 30.
- There is no confounding variable in our final model.

#### Associative model

We also performed an associative model of clinical factors such as age (over 60 years), body mass index (over 30) and the consumption of toxic habits to estimate the increased risk of these clinical factors with the presence of chronic neuropathy.

- Age over 60 years: (OR = 2.46; CI95 1.23-4.91). A patient over 60 years of age, compared to a patient who is not, all other variables being equal, has a 2.46-fold increased risk of chronic CIPN. In the population this risk would be between 1.23-4.91 times higher with 95% confidence.
- BMI greater than or equal to 30: (OR = 2.23; 95%CI 0.95-5.23). A patient with a BMI greater than or equal to 30, compared to a patient without, all other variables being equal, has a 2.23-fold increased risk of chronic CIPN. In the population this risk would be between 0.95-5.23 times higher with 95% confidence.
- Toxic habits: (OR = 2.79; CI95 1.34-5.83). A patient who smokes and/or drinks, compared to a patient who has neither of these habits, all other variables being equal, has a 2.79 times higher risk of developing chronic CIPN. In the population this risk would be between 1.34-5.83 times higher with 95% confidence.

#### Predictive model. risk score

Subsequently, we developed a predictive model and a risk score to help us know which patients are more likely to develop chronic neuropathy, to be able to use it as a preventive model in the future. We considered the following parameters:

$$p\;\left(\text{chronic CIPN}\right)=1\;/\;\left(1+e^{-z}\right)$$

$$z=-0.79+0.898\;-\;\left(\text{Over }60\text{ years}\right)+0.801\;-\;\left(\text{BMI greater than or equal to }30\right)+1.028\;-\;\left(\text{Toxic habits}\right).$$

Each risk factor would be replaced by 1 if you have it, or by 0 if you do not have it.

Looking at the AUC-ROC curve we can see that our model has a statistically significant good prediction, such that in 70.4% of couples where one had chronic CIPN and the other did not. This model would assign a higher probability of developing CIPN to the one with CIPN.

#### Paclitaxel sample, clinical and genetic factors

The statistically significant association between the development of chronic CIPN and chemotherapy drug was only found in patients treated with paclitaxel. Therefore, only these patients were selected for inclusion of genetic polymorphisms in the multiple analyses.

The following results were obtained in the univariate analysis.

- C1236T (C/C genotype): 2.78-fold increased risk of chronic CIPN (p:0.038).
- G2677 T/A: No statistically significant differences were found.
- C3435T (T/T or T/C genotype): 3.46-fold increased risk of chronic CIPN (p:0.029).

#### Associative model

- Age over 60 years: (OR = 3.89; CI95 1.21-12.46). A patient over 60 years of age, compared to a patient who is not, all other variables being equal, has a 3.89 times higher risk of developing chronic CIPN. In the population this risk would be between 1.21-12.46 times higher with 95% certainty.
- Toxic habits: (OR = 4.05; 95%CI 1.2-13.7). A patient who smokes and/or drinks, compared to a patient who has neither of these habits, all other variables being equal, has a 4.05 times higher risk of developing chronic CIPN. In the population, this risk would be between 1.2-13.7 times higher with 95% confidence.
- Cardiovascular risk factors: (OR = 6.86; IC95 1.21-38.97). A patient with AHT and/or DM and/or dyslipidaemia, compared to a patient with none of these factors, all other variables being equal, has 6.86 times the risk of developing chronic CIPN. In the population, this risk would be between 1.21-38.97 times higher with 95% confidence.
- C1236T: (OR = 4.32; IC95 1.41-13.26). A patient with the C/C genotype of the C1236T polymorphism of the ABCB1 gene, compared to a patient without it, all other things being equal, has a 4.32-fold increased risk of developing chronic CIPN. In the population, this risk would be between 1.41-13.26 times higher with 95% certainty.

#### Predictive model. risk score

$$p\;\left(\text{chronic CIPN}\right)=1\;/\;\left(1+e^{-z}\right)$$

$$z=-1.41+1.36\;-\;\left(\text{over }60\text{ years}\right)+1.4\;-\;\left(\text{Toxic habits}\right)+1.93\;-\;\left(\text{CVRF}\right)+1.46\;-\;\left(C1236T\right)$$

Each risk factor would be replaced by 1 if it is present, or by 0 if it is not present.

## Discussion

Chemotherapy-induced peripheral neuropathy is the most frequent neurological complication of cancer treatment and probably the most common toxic neuropathy in our setting. In general, it is estimated that 30%–40% of all patients treated with chemotherapy agents develop peripheral neurotoxicity (Wolf et al., 2008). However, incidences of up to 70% have been reported with the drugs paclitaxel and oxaliplatin during the first month of treatment (Mielke et al., 2006; Grothey, 2005). Although the prevalence of CIPN decreases over time, there are patients who may persist with CIPN 6 months after the end of treatment (Ibrahim and Ehrlich, 2021).

This entity constitutes an important clinical condition in oncology patients, limiting their quality of life due to its diagnostic and management difficulties. In most cases, it requires dose reductions in treatment and even discontinuation of treatment, thus affecting response rates and overall survival (Velasco and Bruna, 2010).

There are two main types of risk factors that can help us to predict and try to prevent it. On the one hand, there are constitutive or innate factors in the patient, such as Single Nucleotide Polymorphisms (SNPs) of genes that influence the metabolism or elimination of neurotoxic drugs (Zhong et al., 2019).

On the other hand, environmental or acquired factors can condition the appearance of CIPN: age, body mass index, the consumption of toxic habits or some cardiovascular diseases (such as arterial hypertension or diabetes mellitus) (Mielke et al., 2006).

Acute CIPN may be associated with the presence of polymorphisms in the ABCB1 gene; whether this pathway is also involved in the chronic entity is unknown. According to the literature, there are several contradictory studies (Zhong et al., 2019).

Öfverholm et al., in 2010 stated that they found no association of *G2677 T/A* and *C3435T* polymorphisms with CIPN in patients treated with paclitaxel (Boora et al., 2016). However, their sample consisted of only 36 patients and lacked a control group. In the same year, Rizzo et al. conducted another study that also included patients treated with docetaxel, but they did not have a control group and no association was found (Rizzo et al., 2010). Tanabe et al., in 2017 defined CIPN from grade 2 onwards, and in an exclusively Asian sample, where they also found no association with ABCB1 C1236T, G2677 T/A or C3435T (Tanabe et al., 2017).

In our NeuroPredict study, not only are the genotypes of each polymorphism analysed individually, but a three-way analysis is performed in a grouped and segmented manner by drug.

In contrast, however, there are other studies that support our results. For example, Tanabe et al. in the same 2017 study analysed a subgroup of patients older than 60 years where they did find differences for the CC genotype of C1236T, as in our study (Tanabe et al., 2017). In 2016, Kus et al. found an increased risk of CIPN of greater than or equal to grade 2 in patients with TT genotype of the *C3435T* polymorphism (Kus et al., 2016).

The relevance of the polymorphisms as predictors of CIPN risk for paclitaxel but not for oxaliplatin is probably due to the existence of other drug metabolisation pathways.

The exact mechanism of peripheral neuropathy induced by platinum-based chemotherapeutics is not yet fully understood; however, it appears that their antitumour mechanisms are responsible for the neurotoxic effect, as chemotherapeutics induce numerous changes in the structure or function of neuronal and glial cells. Chemotherapeutic agents induce various alterations in intracellular organelles (mitochondria), membrane receptors and ion channels, followed by alterations in intracellular homeostasis, signaling and neurotransmission, all of which can lead to neuroinflammation, DNA damage and axonal degeneration (Sałat, 2020).

There is evidence for a role of the glutathione-S-transferase P1 (GSTP1) pathway in antineoplastic resistance. It is a detoxifying enzyme that plays a key role in maintaining cellular integrity and protecting DNA from cell-damaging molecules, inactivating a wide variety of carcinogens or stress-induced toxic intermediates by catalysing their conjugation to reduced glutathione and facilitating its secretion through the liver (Snozek, 2012). Numerous chemotherapeutics, including etoposide, doxorubicin, cisplatin, carboplatin and oxaliplatin, are substrates of GSTP1 (Kishi et al., 2004).

On the other hand, paclitaxel induces altered calcium signalling, neuropeptide and growth factor release, mitochondrial damage, and reactive oxygen species formation. Recent studies also suggest a role for matrix metalloproteinase 13 (MMP-13) in neuropathy. These various changes may be secondary to paclitaxel-induced alteration of microtubule transport (Mielke et al., 2006).

These different metabolism pathways may explain why we do not draw the same conclusions in our results, differentiating the association of ABCB1 polymorphisms with paclitaxel and not with oxaliplatin.

Regarding clinical risk factors, a 2014 meta-analysis and systematic review included neuropathy at baseline, smoking, abnormal creatinine clearance (CrCl) and specific sensory changes during chemotherapy (Seretny et al., 2014). In relation to CrCl rates, the information in our study is likely to be limited because this information is often not available in the patient’s medical record.

A more recent meta-analysis from December 2021 includes duration of drug infusion, initial neuropathy, age, gender, smoking history, renal dysfunction (low ClCr), lifestyle factors and genetic predisposition (Gu et al., 2021). We found it difficult to include the amount of physical exercise performed by patients in our database, as this is not usually reflected in the anamnesis, so we had to eliminate this variable from our study.

A cohort study of 333 participants also conducted in 2021 associated greater severity of CIPN with participants with lower pre-treatment Hb and higher BMI, as well as with older patients and among women (Snozek, 2012). In our sample, there was little heterogeneity in relation to Hb levels, most of them were in the normal range, so, perhaps because of this bias, no association with the development of toxicity was found.

The main difference of this study with respect to others already carried out is fundamentally the definition of the concept of chronic CIPN, which persists despite having finished treatment more than 6 months ago and which is the one that most limits the quality of life of patients. In addition, we included a control group that allows us to compare multiple risk factors for the same pathology and provides us with solid differences under equal conditions for the rest of the variables.

The results of our study raise the possibility that interventions aimed at modifying toxic risk habits and weight control, especially in those patients with risk genotypes, may be a useful strategy in the prevention of chronic CIPN. This needs to be confirmed in future prospective randomised studies.

## Conclusion

Genetic polymorphisms of ABCB1 (*C1236T* and *C3435T*) are associated with chronic chemotherapy-induced peripheral neuropathy in patients treated with paclitaxel. They are not associated in patients treated with oxaliplatin. More specifically, the C/C genotype for *C1236T*, as well as T/T and T/C for *C3435T* are associated with increased risk of chronic chemotherapy-induced peripheral neuropathy in paclitaxel-treated patients. Moreover, there are other factors like age over 60, BMI over 30, the presence of toxic habits such as smoking or alcohol and cardiovascular risk factors such as DM, hypertension or dyslipidaemia which are associated with the development of this toxicity. A score based on BMI, age, and toxic habits for a population of patients treated with oxaliplatin and paclitaxel predicts with specificity the likelihood of developing chronic chemotherapy-induced peripheral neuropathy. A score based on age, toxic habits, cardiovascular risk factors and ABCB1 *C1236T* polymorphism, for a population of patients treated with paclitaxel, predicts with specificity the probability of developing chronic chemotherapy-induced peripheral neuropathy. Nevertheless, more prospective studies are needed to analyse determinants that can help us predict the risk of chemotherapy-induced peripheral neuropathy and thus be able to predict or even treat this toxicity to improve the quality of life of patients.

## Data Availability

The original contributions presented in the study are included in the article/Supplementary Material, further inquiries can be directed to the corresponding author.
